# Diversity of birds in eastern North America shifts north with global warming

**DOI:** 10.1002/ece3.410

**Published:** 2012-11-06

**Authors:** Kenneth W McDonald, Christopher J W McClure, Brian W Rolek, Geoffrey E Hill

**Affiliations:** 1 Ecological Services, U.S. Fish and Wildlife ServiceCookeville, Tennessee, 38501; 2 Department of Biological Sciences, Auburn UniversityAuburn, Alabama, 36849

**Keywords:** Breeding Bird Survey, climate change, diversity, spring temperature, trans-Gulf migrant

## Abstract

The distribution of diversity along latitudinal and elevation gradients, and the coupling of this phenomenon with climate, is a pattern long recognized in ecology. Hypothesizing that climate change may have altered this pattern over time, we investigated whether the aggregate of reported northward shifts of bird ranges in North America is now detectable in community-level indices such as richness and diversity. Here, we report that bird diversity in North America increased and shifted northward between 1966 and 2010. This change in the relationship of diversity to the latitudinal gradient is primarily influenced by range expansions of species that winter in the eastern United States as opposed to species which migrate to this area from wintering grounds in the tropics. This increase in diversity and its northward expansion is best explained by an increase in regional prebreeding season temperature over the past 44 years.

## Introduction

Diversity that declines along latitudinal gradients from the tropics to the poles is one of life's most recognized patterns, and this pattern is strongly correlated with climate (Willig et al. 2003; Currie et al. 2004). Moreover, climate variables such as temperature are highly predictive of these patterns, especially on sufficiently large scales where habitat and contemporary climate coincide to produce these spatial patterns of diversity (Gonzalez-Taboada et al. 2007; Tello and Stevens 2010; Jetz and Fine 2012). Given the relationship between diversity and climate along latitudinal gradients, it is possible climate change will alter the relationship between spatial patterns of diversity and latitude.

This possibility presents unique challenges for conservation. Specifically, when species' habitats exist on islands, atop mountains, as remnant patches, or under other circumstances which constrain the ranges of species, plants, and animals will be unable to respond to changes in climate via range shift. As a consequence, entire communities of organisms may become endangered as the global mean temperature warms over time. Because of the immediacy of the threat to such communities, much of the literature regarding the effects of climate change on animal and plant communities has focused on loss of diversity in isolated habitat patches (Loarie et al. 2009; Sheldon et al. 2011).

**Figure 1:**
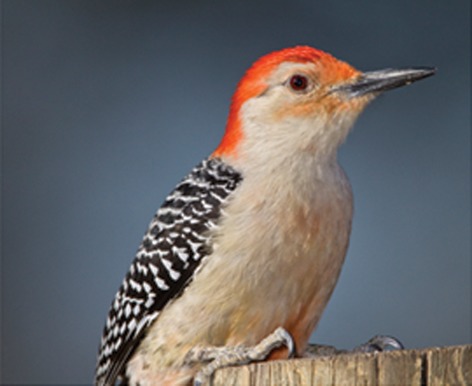
The red-bellied woodpecker (*Melanerpes carolinus*) is an example of a species whose range has expanded northward over time.

In contrast, in the absence of barriers to dispersal, organisms are predicted to respond to rising temperatures by shifting ranges poleward or upward in elevation (Martinez-Meyer 2005; Kearney et al. 2010; Fig. 1). Indeed, in both Europe and North America, many species including butterflies (Parmesan et al. 1999), birds (Parmesan and Yohe 2003; Hitch and Leberg 2007), and plants (Kelley and Goulden 2008; Chen et al. 2011) are reported to be expanding their ranges upward in elevation or toward the poles. Concurrently, climate change has also been implicated in regional increases in community-level indices, such as species richness, which may be a consequence of high-diversity communities shifting into comparatively low-diversity communities at higher latitude or elevation (Menendez et al. 2006; Wilson et al. 2007; Devictor et al. 2008; Hidding and ter Hofstede 2008; Davey et al. 2011).

Climate-induced changes in community-level indices, such as richness or diversity, can also come about because climate change may affect species differently. For example, species able to “track” climate change have been found to advantageously advance nesting dates, survivorship, or increase reproductive success (Frederiksen et al. 2004; Moe et al. 2009; Cleland et al. 2012). Were a similar phenomenon occurring within breeding bird communities in the eastern United States, one would expect similar changes to be detectable in diversity indices of species wintering in the eastern United States (Nearctic species), but not among species which winter in the Neotropics but breed in the Nearctic (Neotropical migrants).

Specifically, we hypothesized the aggregate effect of poleward range expansions by individual species would lead to an increase in breeding bird diversity in North America. We hypothesized a similar increase in diversity within and across communities would occur among Nearctic species but not Neotropical migrants. We also hypothesized that change in prebreeding season temperature (winter or spring) would best explain progressive northward shifts in diversity. We did not hypothesize relationships between richness and diversity among Neotropical migrant and regional temperatures in the eastern United States would be as strong. However, since these latter species do not winter in the eastern United States, it seemed possible that temperature change on larger scales (hemispheric or global) might better explain any observed shifts in diversity among these species, should they too be affected. However, because rising temperatures in Neotropical wintering grounds seemed unlikely to cue northward range expansions on Nearctic breeding grounds, we predicted this was not likely the case and that change in temperatures in the eastern United States would be more explanatory.

Toward understanding whether the aggregate impact of species range shifts among birds have become detectable in community-level indices in North America, we analyzed diversity along Breeding Bird Survey (BBS) routes in the eastern United States between 1966 and 2010. Broadly, we predicted global warming over the past 40 years, facilitating range expansions of individual bird species in the absence of barriers to dispersal, will have caused mean species diversity in the eastern United States to increase. We predicted this would be most strongly explained by northward shifts in diversity over this period. Also, because Nearctic species are present during the prebreeding months, we predicted this group would increase in diversity and richness measures within routes at northern latitudes, as well as across routes at all latitudes. Because Neotropical migrants winter outside of this region and are presumably not present to exploit changes in prebreeding season temperature to their advantage, we predicted diversity and richness of these species would either decrease or remain unchanged. We predicted change in diversity and location of areas of high diversity within and across communities would be explained by changing prebreeding season temperature in the eastern United States rather than on larger spatial scales; and that changing prebreeding season temperature in this region would explain change in species richness among Nearctic species but not Neotropical migrants.

## Materials and Methods

**Figure 2:**
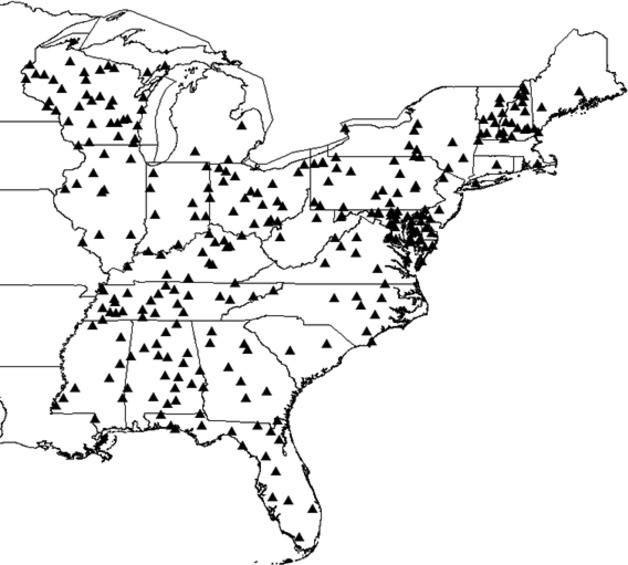
The locations of the 291 Breeding Bird Survey routes evaluated in the course of this study. These routes were surveyed in both 1966 and 2010, and contained at least 15 intervening years of records.

We used bird survey data from BBS routes to test for changes in diversity of avian communities between 1966 and 2010 in the eastern United States. Although methodology employed by the BBS has been criticized (Bart et al. 2004; Kéry and Schmid 2004), the BBS remains a valuable index of bird population levels and distributions within North America (Johnson 2008). Data for all species and routes available through the FTP site were obtained from the USGS Patuxent Wildlife Research Center (USGS 2011). From these data, we identified 291 routes in the eastern United States, all occurring between the Mississippi River and the Atlantic Ocean, from Maine to Florida, that were surveyed in both 1966 and 2010 with at least 15 years of intervening records (Fig. 2). We found 107 species of birds had been surveyed along these routes at some point during the record of observation.

For each BBS route, we calculated Inverse Simpson's Index of Diversity (1 – the alpha diversity measure of species richness within a community; hereafter referred to as “diversity”). Diversity estimates for individual routes were then averaged as a measure of overall, across-route diversity, which were used to calculate the change in diversity across years. We also investigated annual change in diversity across latitudes by calculating the average annual latitude of the 80% diversity contour across the eastern United States. This contour represents latitude at which there is an 80% probability of selecting a different species on a second sampling, and because contours of all other diversity increments behaved similarly over time, the 80% diversity contour served as an arbitrary index of the yearly latitudinal distribution of diversity in our study. We then calculated the yearly change in the latitude of the 80% diversity contour as a proxy for changes in the spatial distribution of bird diversity in the eastern United States. Finally, from the BBS data set, we calculated annual, mean richness, and diversity of year-round Nearctic species and Neotropical migrant species across routes (Shackelford et al. 2005), between the first and last decades of the record (1966–1975 and 2001–2010).

For analysis of all metrics (annual mean Simpson's diversity across communities, mean latitude of the 80% contour, Nearctic species richness, and Neotropical migrant richness) with climate, we obtained monthly mean temperatures for 66 weather stations spanning the eastern United States from the United States Historical Climatology Network data access portal and we used these data to calculate change in winter temperature between years (Menne et al. 2011). We additionally included temperature anomalies on larger geographic scales which might be more representative of temperature anomalies affecting species wintering in both Nearctic and Neotropical regions. For this purpose, data for Northern Hemispheric Temperature Anomaly and Global Land/Sea Surface Temperature Anomaly were obtained through the National Oceanic and Atmospheric Administration Climate Indices: Monthly Atmospheric and Ocean Time Series portal (Hansen et al. 2010). These were used as competing hypotheses for drivers of northward shifts in over-all breeding bird diversity over time. Because more specific regional data was available for the eastern United States, we used change in winter, spring, and summer temperature in this region as competing hypotheses for increases in mean species richness/BBS route.

We used Generalized Additive Models to determine whether diversity across communities and the latitude of the 80% diversity contour each increased over time. These models fit a thin plate spline – a smoothed, two-dimensional surface – through the data. We used Generalized Cross Validation to adjust degrees of freedom and reduce the risk of over-fitting the spline to the data using the mgcv package (Wood 2006) in the R statistical programming environment (R Development Core Team 2002).

We also used Box-Jenkins (Box and Sato 1970) time series analysis to test whether change in diversity across communities and change in the latitude of the 80% diversity contour were each correlated with changing temperature and temperature departures from average on different spatial scales. We examined temporal autocorrelation in the two time series by comparing an autoregressive model (AR-1) with a null model using generalized least squares. We compared the null model and the autoregressive model for each analysis using Akaike's Information Criterion corrected for small sample size (AIC_*c*_, Burnham and Anderson 2002). For the yearly change in diversity, the null model received a lower AIC_*c*_ value (−344.74) than the autoregressive model (−343.48). We therefore used linear regression to analyze the yearly change in diversity. For the yearly change in the average latitude of the 80% diversity contour, the autoregressive model received a lower AIC_*c*_ value (49.95) than the null model (52.65), indicating temporal autocorrelation. We therefore used autoregressive models to examine changes in the average latitude of the 80% diversity contour. Finally, for the yearly change in the Nearctic species richness observed per route, the autoregressive model received a lower AIC_*c*_ value (115.68) than the null model (125.22), so we used the autoregressive model to examine this as well.

We then built models relating changes in diversity and latitude of the 80% diversity contour to regional, hemispheric, and global climatic variables (Table 1). We also hypothesized that an overall increase in diversity across eastern North America might be explained by a northward shift in the latitude of the 80% diversity contour. We therefore built an additional model relating the change in overall diversity to the change in latitude of the 80% diversity contour. Finally, we predicted that change in mean Nearctic species richness observed per BBS route would increase over time, and this would be attributable to changing prebreeding season temperature (spring or winter). We ranked and compared models for diversity and latitude of the 80% diversity contour separately using AIC_*c*_. We considered models that were ΔAIC_*c*_ >2 from the null and had *w*_i_ >0.6 as more informative than random, and covariates within models ΔAIC_*c*_ ≤2 and with confidence intervals not overlapping zero, as having possible additional informative value (Chandler et al. 2009).

**Table 1:** Change in diversity

Predictors	*df*	AIC_c_	ΔAIC_c_	*w*_i_
Δ Latitude of the 80% diversity contour	3	−348.31	0.00	0.65
Δ Eastern U.S. winter temperature	3	−345.14	3.17	0.13
Null	2	−344.74	3.57	0.11
Global temperature anomaly	3	−342.55	5.76	0.04
Northern hemisphere temperature anomaly	3	−342.42	5.88	0.03
Eastern U.S. temperature anomaly	3	−342.42	5.88	0.03

Increasing diversity across 291 BBS routes over 44 years is best explained by the northward shift of the contour representing an 80% probability of drawing a different species on a second sampling, while the northward shift of the contour representing an 80% probability of drawing a different species on a second sampling is best explained by increasing winter temperature over the eastern United States. Models are ranked by their AIC_c_ value (Akaike's Information Criterion adjusted for small sample size). ΔAIC_c_ is the difference between each model's AIC_c_ value and the AIC_c_ value of the best-supported model and *w*_i_ is the probability that a model is the best model within the model set, and *df* is the degrees of freedom for each model.

## Results

**Figure 3:**
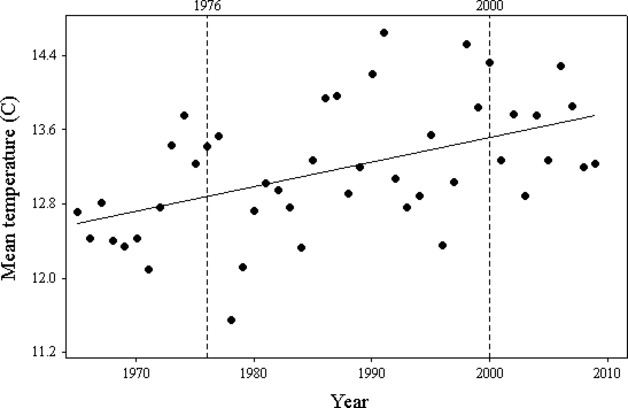
Average annual temperature over the eastern United States from 1966 to 2010. From the first decade in the BBS record to the most recent, average annual winter, spring, and summer temperature increased by ∼1.3°C (1966–1976 vs. 2000–2010, two-sample *t*-test, *P* < 0.0001). This change occurred across 66 stations that participate in the United States Historical Climatology Network in the eastern United States, between the Mississippi River and the Atlantic Ocean, from the St. Lawrence River to the Gulf of Mexico.

**Figure 4:**
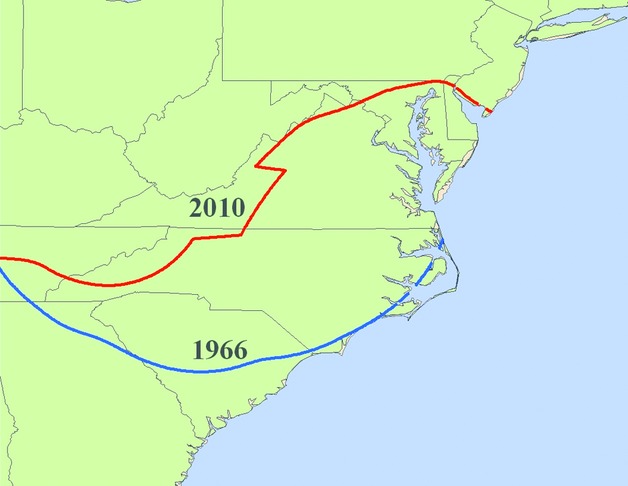
Species diversity shifted northward between 1966 and 2010. West of the Appalachian Mountains, the northward shift in diversity appears muted possibly because of the effects of topography in this region, where elevation generally increases on a west-to-east axis.

**Figure 5:**
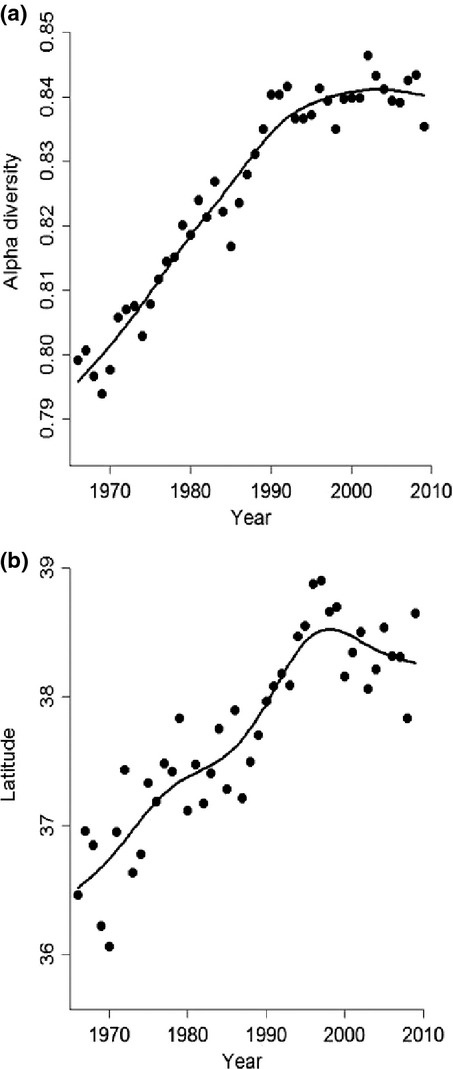
(a) A thin plate spline of alpha diversity by year. From 1966 to 2010, diversity across BBS routes increased significantly; (b) A thin plate spline of latitude of Simpson's Diversity 80% contour by year. From 1966 to 2010, this arbitrary measure of the spatial relationship between diversity and latitude shifted northward.

**Figure 6:**
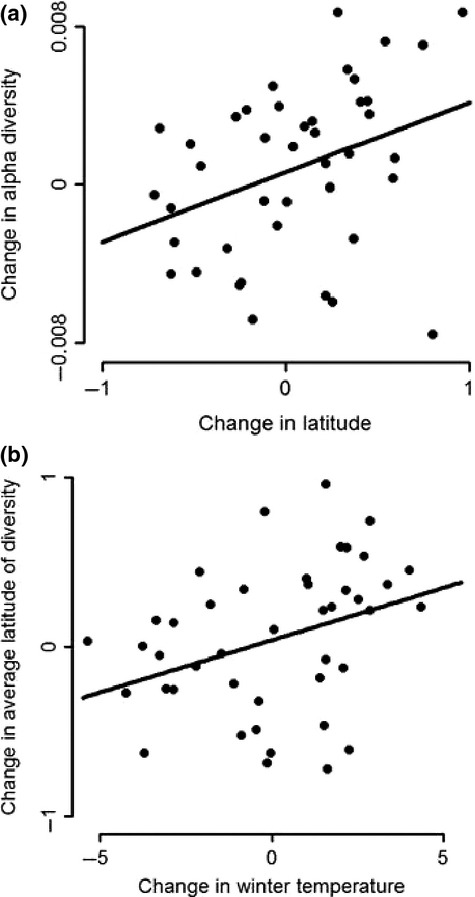
Increasing diversity across communities is explained by progressively northward increases of diversity within communities. (a) Increasing alpha diversity across communities is explained by the change in the average latitude Simpson's Diversity 80% contour (AIC*w* = 0.65). (b) Change in the average latitude of the Simpson's Diversity 80% contour is best explained by increasing winter temperature within the eastern United States (AIC*w* = 0.77).

In the eastern United States, over a period of 44 years, average annual temperature increased by ∼1.3°C (Fig. 3). During this same period breeding bird diversity both increased and moved northward. The thin plate splines for both diversity and latitude of the 80% diversity contour were significant (*P* < 0.001) and explained 94.3% and 83.4% of the deviance, respectively (Figs. 4, 5a and b). The observed change in diversity was best explained by northward shifts of breeding bird diversity that occurred between 1966 and 2010 (*b* = 0.004, *t* = 2.45, *P* = 0.02, Table 1, Fig. 6a). Our analysis also found that the northward shift of diversity within communities was significantly, positively explained by increasing regional winter temperatures that occurred throughout the eastern United States during the period of observation (*b* = 0.06, *t* = 2.55, *P* = 0.01, Table 1, Fig. 6b).

**Figure 7:**
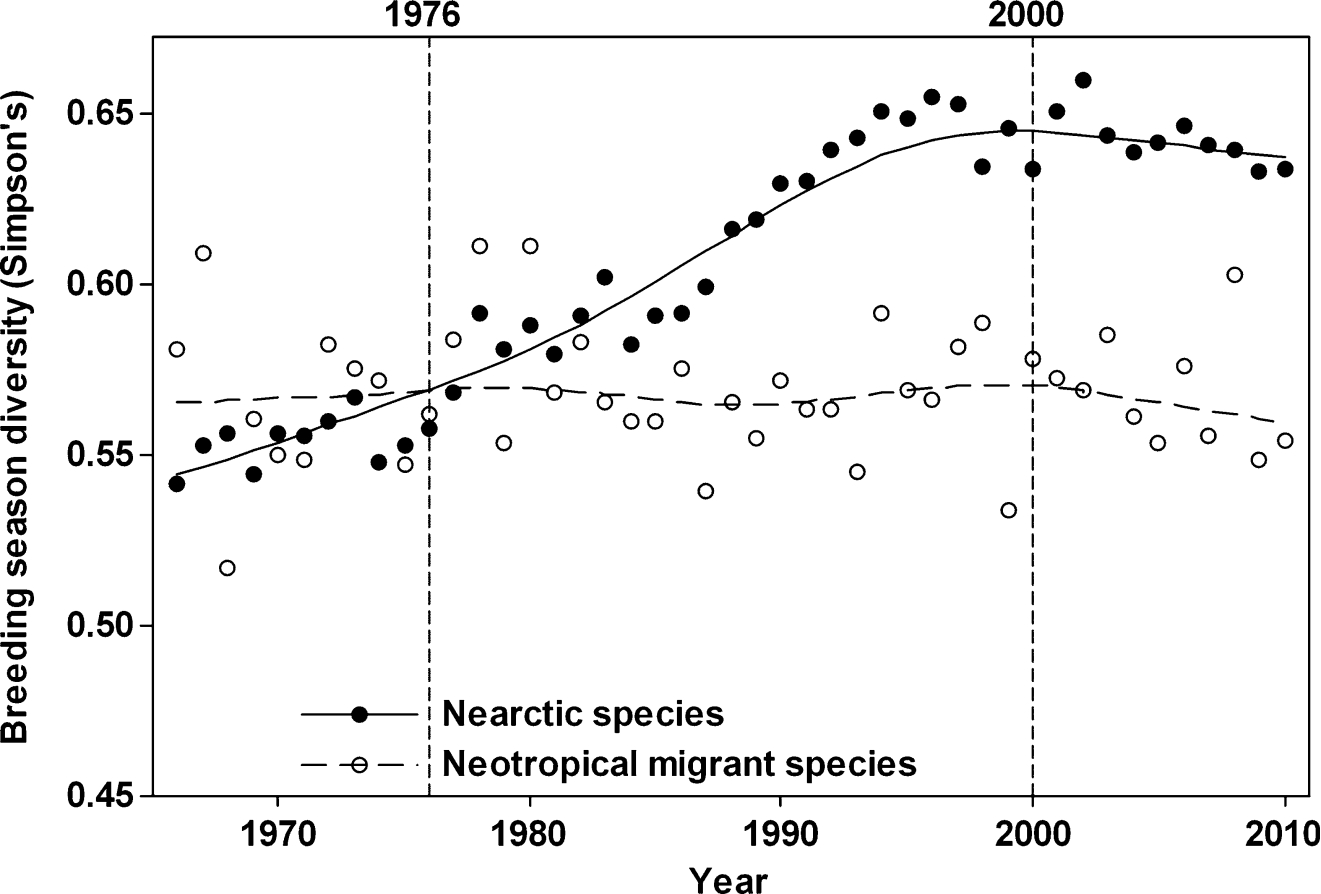
Scatterplot of average annual species diversity across BBS routes, among Nearctic bird species (those which winter in the eastern United States), by year from 1966 to 2010, and Neotropical migrants. The mean number of Nearctic species increased by ∼2.4 species per route between the first and last decade of the record (two-sample *t*-test, *P* < 0.0001), while the mean number of Neotropical migrant species (defined as species identified as typically wintering in the Neotropics but breeding in the Nearctic United States) observed by route did not change (two sample *t*-test, *P* = 0.133).

**Figure 8:**
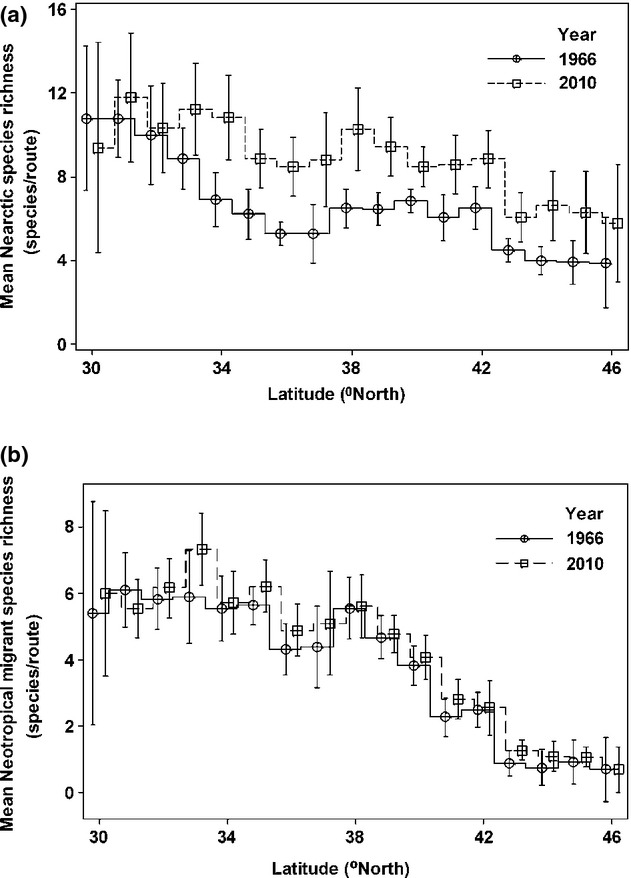
(a) Interval plot of mean Nearctic species richness by latitude, between 1966 and 2010, observed across BBS routes. Species richness of Nearctic species increased uniformly across latitudes by ∼2.4 species, between 1966 and 2010. (b) Interval plot of Neotropical species richness by latitude, between 1966 and 2010, observed across BBS routes. Species richness of Neotropical migrants did not significantly change across latitudes between 1966 and 2010.

We also found mean Nearctic species richness across routes increased by ∼2.4 species over the same period, and this occurred uniformly across latitudes. This was matched by a significant increase in Nearctic species diversity from the first decade of the record to the last (1966–1975 vs. 2001–2010; two-tailed *t*-test, *P* < 0.0001, Figs. 7, 8a). Mean Neotropical migrant species richness across routes did not increase at any latitude and there was no significant increase in the average diversity of Neotropical migrants between the first and last decades of the survey (two-tailed *t*-test, *P* < 0.133, Figs. 7, 8b). Finally, our analysis of changing winter, spring, and summer temperatures within the eastern United States found that changing spring temperature, specifically, strongly predicted change in Nearctic species richness (Table 2).

**Table 2:** Change in average number of nearctic species × route

Predictors	*df*	AIC_c_	ΔAIC_c_	*w*_i_
Δ Spring temperature	3	118.27	0.00	0.824
Δ Summer temperature	3	123.01	4.664	0.080
Δ Winter temperature	2	123.46	5.115	0.064
Null	3	124.85	6.501	0.032

Increases in mean Nearctic species richness observed per BBS route is best explained by increasing spring temperature. Models are ranked by their AIC_c_ value (Akaike's Information Criterion adjusted for small sample size). ΔAIC_c_ is the difference between each model's AIC_c_ value and the AIC_c_ value of the best-supported model and *w*_i_ is the probability that a model is the best model within the model set, and *df* is the degrees of freedom for each model.

## Discussion

Using BBS data, we found mean diversity of bird species both increased and shifted north in the eastern United States over the past 44 years. Likewise, we found an increase in Nearctic species richness and diversity, but no change in Neotropical migrant species richness and diversity. Increasing prebreeding season temperatures, winter for diversity as a whole and spring for species richness among Nearctic birds had the most explanatory power for these phenomena, respectively. Changing hemispheric and global mean temperature during the nonbreeding season, while correlated with change in regional temperature, did not more parsimoniously explain the pattern of change observed.

Broadly, that changing prebreeding season temperatures appear to be strong predictors for the changes in Nearctic diversity is hardly novel; for the potential for temperature to have effects on the biology of breeding birds have been previously and widely reported elsewhere (McCarty 2001; Rosenzweig et al. 2008). For instance, temperature during winter or spring months is known to affect species communities by temporally shifting peak net primary productivity forward in time, or by affecting the magnitude of peak net primary productivity over the course of the breeding season (Durant et al. 2007; Yom-Tov and Geffen 2011). It has also been shown that when increases in net primary productivity occur, they typically and disproportionately occur in ecosystems located in historically colder regions, where increases precipitation and temperature have resulted in increased growing season length (Rustad et al. 2001; Huntington et al. 2009). Here, we note it is within communities at such latitudes the majority of change reported seems to have occurred; again a result that is in itself unsurprising.

However, net diversity across communities in some regions did not change as quickly as it did in others. Specifically, we note the greatest regions of northward shift occurred where elevation was relatively homogenous, for example, along the coastal plains and piedmont of the eastern United States. Where areas of rapid changes in elevation were encountered, it seems change in diversity may have been constrained by the presence of elevational gradients. This may explain the apparent “wrapping” of diversity along the Appalachian escarpment in areas such as Virginia and North Carolina, and the muted change observed where the 80% contour traveled the spine of an elevation gradient reaching from the Mississippi River to the Cumberland Plateau in Tennessee. However, because we did not anticipate change along elevation gradients would be detectable at this scale, we did not assess whether shifts in diversity also occurred along elevation gradients, explicitly. Thus, we can only make an informed inference of such an effect from the spatial pattern of change observed, here. This said, we remain confident that although elevation gradients may serve as an alternative vector for diversity responding to climate change in some regions, the dominant, or at the least analogous narrative, is that of poleward shifting diversity.

We also infer from our results that increasing temperatures across the eastern United States during the months prior to breeding are likely advantaging Nearctic species wintering in this region. So although Neotropical migrants may not have been adversely impacted by increasing prebreeding season temperatures, it would appear they may be becoming relatively less represented in the BBS simply because their abundance within communities is not growing as quickly as that of Nearctic species. Had Neotropical migrants been adversely impacted and in decline because of changing temperature, one would have expected a subsequent range contraction to follow, as has occurred among species in decline, broadly (Rodriguez 2002). Such phenomena elsewhere have been found to effect diversity (Lemoine and Bohning-Gaese 2003; Lemoine et al. 2006). However, our analysis found no evidence of decline in Neotropical migrant diversity or richness, and any effect their relative representation may have diversity as a whole is more likely explained by the difference in growth rate of diversity between these two groups rather than by an increase in one and decline in the other.

Finally, although we observe a strong relationship between trends in climate and diversity, we also concede our analysis cannot completely discount other hypothesized factors contributing to shifts in diversity such as changes in land use and habitat fragmentation over the last half-century. However, although it is possible human or natural alterations and fragmentation of the landscape may have had an impact on the distribution of species at smaller scales, it is very difficult to imagine how a myriad of diverse but comparatively localized anthropogenic activities could have established a global latitudinal gradient of diversity and then caused that diversity gradient to slip consistently poleward or upslope over a relatively short period of time spanning 44 years.

In the case of our study, it is additionally difficult to imagine why anthropogenic changes in the landscape would have affected breeding populations of Nearctic species but not Neotropical migrants. In support of this, we note La Sorte and Thompson (2007) found that anthropogenic alterations and fragmentation of landscapes only account for a small percentage of northward latitudinal shifts among species that winter in the United States and southern Canada, and this is an important distinction to make as it were these species that showed the strongest response to warming in this study. Naturally, this does not mean more proximal human alterations and fragmentation of the landscape have no effect on avian diversity. Rather, we simply recognize the more parsimonious explanation for the establishment of the initial, global latitudinal gradient of diversity, and subsequent change in diversity along that gradient, is climate and climate change; a pattern and change in pattern our analysis supports. For this reason, we are confident our results thus reveal – at a community level – the cumulative effects of a warming climate driving the ranges of individual species poleward or upslope.
